# Four Cases of Myeloproliferative Disorders Associated With Down Syndrome: Distinguishing ML-DS From TAM-DS: Distinguishing TAM-DS and ML-DS: Report of 4 Cases

**DOI:** 10.1155/2024/9962512

**Published:** 2024-10-23

**Authors:** Kévin Boumeghar, Sylvie Daliphard, Nimrod Buchbinder, Catherine Boutet, Dominique Penther, Pascaline Etancelin, Julien Bourgain, Gérard Buchonnet, Elsa Bera, Victor Bobée

**Affiliations:** ^1^Rouen University Hospital, Department of Biological Hematology, Rouen 76000, France; ^2^Rouen University Hospital, Department of Pediatric Hematology, Rouen 76000, France; ^3^Centre Henri Becquerel, Department of Genetic Oncology, Rouen 76000, France

**Keywords:** down syndrome, GATA1, ML-DS, TAM-DS, transient abnormal myelopoiesis

## Abstract

Down syndrome (DS) is defined by an extra copy of chromosome 21 and confers an increased susceptibility to hematological disorders. Transient abnormal myelopoiesis (TAM) and myeloid-leukemia associated with Down syndrome (ML-DS) are two conditions that need to be accurately diagnosed to provide appropriate management. Both TAM and ML-DS are characterized by proliferation of megakaryoblasts carrying a mutation in the GATA1 gene. Here, we report four cases with educational significance, highlighting typical diagnostic features that facilitate the differentiation between these two conditions, thereby assisting clinicians and medical laboratory professionals in effectively managing and monitoring these patients.

## 1. Introduction

Down syndrome (DS), also known as trisomy 21, is the most common chromosomal abnormality in live-born infants, characterized by an extra copy of chromosome 21. This genetic condition confers an increased susceptibility to a variety of hematologic disorders, including transient abnormal myelopoiesis (TAM) and myeloid leukemia associated with DS (ML-DS). TAM is a unique phenomenon observed in newborns with DS. It typically presents during the neonatal period as a transient clonal expansion of blasts, often remaining clinically silent, and spontaneously regressing within the first weeks of life. Despite its transient nature, close monitoring is needed as clonal expansion toward Myeloid-Leukemia associated with Down syndrome occurs in the first years of life in 20%–30% of the cases [1]. Myelodysplastic syndrome (MDS) and acute myeloid leukemia (AML), now jointly classified as ML-DS in the WHO classification [2], typically manifest before the age of five with a unique propensity for megakaryoblastic differentiation and require intensive chemotherapy. AML and ML-DS arise from the same underlying genetic predisposition and overlapping features can lead to diagnostic confusion. Through the extensive analysis of four cases including morphology, immunophenotyping, cytogenetic, and molecular study, we delineate distinctive characteristics that facilitate the differentiation between TAM-DS and ML-DS in daily practice, thereby assisting clinicians and medical laboratory professionals in effectively managing and monitoring these patients.

## 2. Case Presentation

**Figure 1:**
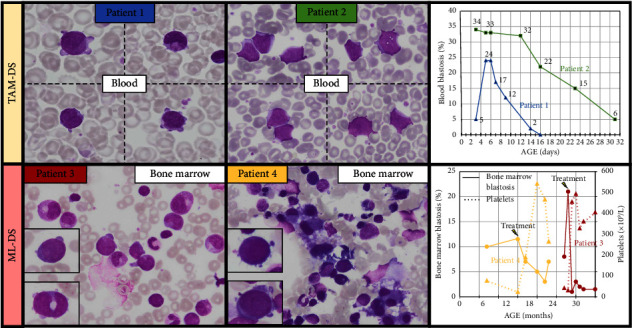
Blast morphology and evolution of blasts over time. Blasts morphology of TAM-DS and ML-DS are characterized by the presence of large cells with round nuclei and “blebs,” irregular protrusions of the cell membrane, altogether suggesting a megakaryoblastic origin. The morphology of blasts in TAM-DS (cases 1 and 2) exhibited no significant differences compared to blasts in ML-DS (cases 3 and 4). In case 3, rare granulations were visible. In case 4, bone marrow analysis revealed dysmegakarocytopoiesis including the presence of micromegakaryocytes (May Grünwald Giemsa, original magnification 1000x). In TAM-DS cases, evolution of blast count in blood is illustrated, showing a decrease after a few days. For ML-DS cases, blast counts in the bone marrow are presented along with platelet counts and their evolution after treatment. ML-DS, myeloid leukemia associated with down syndrome; TAM, transient abnormal myelopoiesis.

Patient one was a boy delivered by caesarean section due to intrauterine growth restriction and abnormal fetal heart rhythm. At 3 days of age, DS was diagnosed through cytogenetic screening. His total blood count was normal with leukocyte count at 16.5 × 10^9^/L. Differential blood count revealed a significant blood blast count of 24%, characterized by large cells with round nuclei, immature chromatin, basophilic cytoplasm, and “blebs”—irregular protrusions of the cell membrane that suggest a megakaryoblastic origin (Figure 1). Immunophenotyping of blood leukocytes was performed, showing CD34+ CD38+ blasts with two myeloid markers (CD33+ CD117+), as well as positivity for CD42b (also known as glycoprotein Ib) and CD61 (glycoprotein IIIa), specific to the megakaryocytic lineage. In addition, the blasts showed positivity for CD36 (glycoprotein IV). A frameshift mutation c.101_102insT, p.S36Lfs∗4 affecting exon two of the *GATA1* gene was identified through Sanger sequencing, resulting in a premature stop codon. The boy remained asymptomatic, and the blood count normalized within 15 days. The diagnosis of TAM-DS was made and a quarterly surveillance checkup was established. The boy is currently 18 months old and still has a normal blood count.

Patient two was a female diagnosed with DS during prenatal screening. Hepatosplenomegaly was observed on the third day of life. The blood count showed significant hyperleukocytosis (38 × 10^9^/L) with no other abnormalities. Blood smear analysis highlighted a blast count accounting for 34% of leukocytes and characterized by large cells, nucleoli and irregular shape (Figure 1). The megakaryoblastic lineage was confirmed through immunophenotyping of blood leukocytes, which showed positivity for CD36+, CD42+, and CD61+ (among other markers: CD34+, CD38+, CD33+, CD117+). A missense mutation, c.1A > G, p.M1V, affecting exon one of the *GATA1* gene was identified by Sanger sequencing, resulting in a substitution of the initiation codon. The blood count normalized within a month, confirming the diagnosis of TAM-DS. The evolution was uneventful, and the girl remains in good health at 18 months.

Patient three, a 2-year-old male with DS, underwent follow-up examinations, including blood analyzes, which revealed isolated severe thrombocytopenia at 42 × 10^9^/L. There were no detectable blast cells in the peripheral blood smear. The clinical examination did not show any abnormalities. These findings prompted a bone marrow aspiration that revealed an initial blast count of 8% with rare dysplastic megakaryocytes. Subsequent aspiration 1 month later indicated an increase to 21% blasts. Most of the blasts exhibited characteristics suggestive of a megakaryoblastic lineage, including large size, round nucleus, and frequent cytoplasmic blebs (Figure 1). Rare granulations were observed in the blasts. Bone marrow immunophenotyping supported the megakaryoblastic phenotype of blasts (CD36+, CD42+, CD61+, CD33+, CD117+, CD34+, and CD38+). The *GATA1* gene was found to harbor the mutation c.97delinsGAAA; p.33delins fs∗6, located in exon two, resulting in a frameshift mutation with a premature stop codon, as identified through Sanger sequencing. In addition to the known trisomy 21, an unbalanced translocation [3, 4] was identified. There was no history of TAM-DS in the first weeks of life. A blood count had been performed at 3 days of life showing transient thrombocytopenia at 32 G/L with no abnormal cells observed on blood smear. The diagnosis of ML-DS was established, and the patient received treatment according to the “ML-DS 2006” protocol, which included the administration of cytarabine, idarubicin, and etoposide, resulting in complete remission. The patient is now 8 years old and has shown a favorable clinical course with no relapses.

Patient four, a 7-month-old female with DS, underwent a follow-up examination. The blood count revealed thrombocytopenia at 77 × 10^9^/L and neutropenia at 0.95 × 10^9^/L. There were no clinical symptoms, and the blood smear examination showed no abnormalities. Given the persistence of cytopenias, a bone marrow aspiration was performed, which revealed 10% blasts displaying megakaryoblastic morphology. In addition, the bone marrow analysis revealed significant dysmegakaryocytopoiesis, including the presence of micromegakaryocytes (Figure 1). Immunophenotyping of bone marrow cells confirmed a megakaryoblastic phenotype with positivity for CD36, CD42, and CD61 (among other markers, CD34+, CD38+, CD33+, and CD117+). An additional trisomy eight was found through cytogenetic studies and high-throughput sequencing identified a *GATA1* c.170_180dupCTGCGGCACTG; p.A61Lfs∗80 mutation with a low variant allele frequency (VAF) of 2%, revealing a frameshift alteration in exon two and a premature stop codon at position 80. The diagnosis of ML-DS was established, leading to the patient receiving treatment as in the “ML-DS 2006” protocol. The patient is currently in complete remission 1 year after completing treatment and is now 3 years old. No blood count was performed at birth and a history of TAM-DS cannot be ruled out.

## 3. Discussion

**Figure 2:**
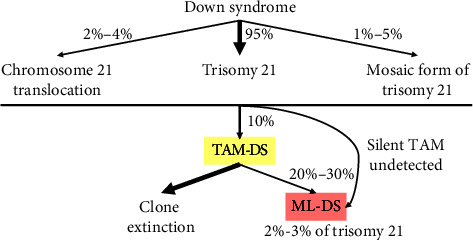
Multistep pathogenesis of TAM-DS and ML-DS. ML-DS, myeloid leukemia associated with down syndrome; TAM, transient abnormal myelopoiesis.

DS confers a higher risk of malignancy. Approximately 10% of neonates with DS experience TAM and around 2%–4% will develop ML-DS before reaching 4 years of age [1, 5, 6] (Figure 2). While the onset periods for TAM and ML-DS are generally nonoverlapping, differentiating them remains crucial and can be challenging for nonspecialist physicians. In this study, we present two cases of TAM-DS and two cases of ML-DS, highlighting the distinguishing features between these two conditions (Table 1). TAM-DS typically manifests during the initial days of life, whereas ML-DS tends to develop after several months. There are usually no cytopenias in TAM-DS, unlike in ML-DS. A substantial proportion of blast cells can be encountered in TAM-DS during newborn blood counts. Conversely, in ML-DS, the presence of blasts in the blood is not always apparent, illustrated by our two cases where bone marrow aspiration was necessary to confirm the diagnosis. The blast morphology and immunophenotyping reveal a megakaryoblastic lineage without specific distinctions between these two conditions, as previously described by other studies [3, 7]. Additional structural cytogenetic abnormalities aside from the trisomy 21 are frequently associated with ML-DS [4, 7]. The *GATA1* mutation is a hallmark feature in both TAM and ML-DS leading to an imbalance of RUNX1, yet it does not provide distinction between these states [5, 7–9].

## 4. Conclusion

Overall, a complete blood count should be performed during the neonatal period in all neonates with DS to identify potential TAM-DS. Confirmation of the megakaryoblastic lineage by flow cytometry should be performed. In addition, it is essential to conduct *GATA1* mutation testing to confirm its involvement and establish it as a diagnostic marker during the follow-up. It is important to note that high-throughput sequencing should be performed if no mutation is detected by Sanger sequencing to ensure sufficient sensitivity [10], as illustrated by case 4 in this study, where Sanger sequencing yielded normal results, and the mutation with a VAF of 2% was only uncovered through high-throughput sequencing. Despite being asymptomatic in most cases, some TAM-DS patients may develop complications such as organ failure (particularly liver disease), leukostasis syndrome, hyperleukocytosis, or disseminated intravascular coagulation. In such instances, low doses of cytarabine are considered. For patients without these complications, the natural decrease in blast cells typically occurs within a few weeks.

When children with DS develop cytopenias, suspicion of progression to ML-DS arises, prompting a bone marrow aspiration and detection of the *GATA1* mutation, regardless of whether there is a known history of TAM. The diagnosis of ML-DS can be established even if the percentage of medullar blasts is less than 20%, a threshold typically considered in AML [5]. In addition to our results, previous studies have found dyserythropoiesis in ML-DS but not in TAM [7]. Moreover, in a large cohort of TAM, MRD positivity assessed by flow cytometry at 3 months of life was found predictive of leukemia development [8]. The challenge in ML-DS then relies in maintaining chemotherapy in the context of DS as patients often have difficulty thoroughly understanding their condition.

Finally, identifying TAM at birth is essential to establish a routine monitoring schedule until the age of five [1, 5, 6] to enable early detection of the reemergence of the dormant clone, characterizing ML-DS.

## Figures and Tables

**Table 1:** Biological presentation of the cases including 2 TAM-DS and 2 ML-DS.

	TAM-DS		ML-DS	
	Patient 1	Patient 2	Patient 3	Patient 4
Age	3 days	3 days	2 years	7 months

Hemoglobin (g/dL)	17.7	14.7	12.2	11.7

Platelets (×10^9^/L)	193	349	42	77

Leukocytes (×10^9^/L)	16.5	38	8.3	4.5

Neutrophils (×10^9^/L)	2.64	11.78	4.9	0.95

Blasts (%)	24	34	0	0

Cytogenetic study	T21	T21	T21 + *t*(5;7)	T21 + T8

GATA1 sequencing	Frameshift Exon 2 Sanger sequencing	Missense Exon 1 Sanger sequencing	Frameshift Exon 2 Sanger sequencing	Frameshift Exon 2 NGS VAF2%

Abbreviations: ML-DS, myeloid leukemia associated with down syndrome; TAM, transient abnormal myelopoiesis.

## Data Availability

All data are available from the corresponding author upon reasonable request.

## References

[1] Kosmidou A., Tragiannidis A., Gavriilaki E. (2023). Myeloid Leukemia of Down Syndrome. *Cancers*.

[2] Khoury J. D., Solary E., Abla O (2022). The 5th Edition of the World Health Organization Classification of Haematolymphoid Tumours: Myeloid and Histiocytic/Dendritic Neoplasms. *Leukemia*.

[3] Brouwer N., Matarraz S., Nierkens S (2022). Immunophenotypic Analysis of Acute Megakaryoblastic Leukemia: A EuroFlow Study. *Cancers*.

[4] Massey G. V., Zipursky A., Chang M. N (2006). A Prospective Study of the Natural History of Transient Leukemia (TL) in Neonates With Down Syndrome (DS): Children’s Oncology Group (COG) Study POG-9481. *Blood*.

[5] Roberts I. (2022). Leukemogenesis in Infants and Young Children with Trisomy 21. *Hematology*.

[6] Baruchel A., Bourquin J. P., Crispino J (2023). Down Syndrome and Leukemia: From Basic Mechanisms to Clinical Advances. *Haematologica*.

[7] van den Akker T. A., Liu Y. C., Liu H (2023). Myeloid Proliferations Associated With Down Syndrome: Clinicopathologic Characteristics of Forty Cases From Five Large Academic Institutions. *Pathobiology*.

[8] Yamato G., Deguchi T., Terui K (2021). Predictive Factors for the Development of Leukemia in Patients With Transient Abnormal Myelopoiesis and Down Syndrome. *Leukemia*.

[9] Gialesaki S., Bräuer-Hartmann D., Issa H (2023). RUNX1 Isoform Disequilibrium Promotes the Development of Trisomy 21-Associated Myeloid Leukemia. *Blood*.

[10] Terui K., Toki T., Taga T (2020). Highly Sensitive Detection of GATA1 Mutations in Patients With Myeloid Leukemia Associated With Down Syndrome by Combining Sanger and Targeted Next Generation Sequencing. *Genes Chromosomes & Cancer*.

